# The Advancing of Zinc Oxide Nanoparticles for Biomedical Applications

**DOI:** 10.1155/2018/1062562

**Published:** 2018-07-05

**Authors:** Jinhuan Jiang, Jiang Pi, Jiye Cai

**Affiliations:** ^1^State Key Laboratory of Quality Research in Chinese Medicines, Macau University of Science and Technology, Macau, China; ^2^Department of Chemistry, Jinan University, Guangzhou, China

## Abstract

Zinc oxide nanoparticles (ZnO NPs) are used in an increasing number of industrial products such as rubber, paint, coating, and cosmetics. In the past two decades, ZnO NPs have become one of the most popular metal oxide nanoparticles in biological applications due to their excellent biocompatibility, economic, and low toxicity. ZnO NPs have emerged a promising potential in biomedicine, especially in the fields of anticancer and antibacterial fields, which are involved with their potent ability to trigger excess reactive oxygen species (ROS) production, release zinc ions, and induce cell apoptosis. In addition, zinc is well known to keep the structural integrity of insulin. So, ZnO NPs also have been effectively developed for antidiabetic treatment. Moreover, ZnO NPs show excellent luminescent properties and have turned them into one of the main candidates for bioimaging. Here, we summarize the synthesis and recent advances of ZnO NPs in the biomedical fields, which will be helpful for facilitating their future research progress and focusing on biomedical fields.

## 1. Introduction

Recently, biomedical nanomaterials have received more concerns because of their prominent biological characteristics and biomedical applications. With the development of nanomaterials, metal oxide nanoparticles show promising and far-ranging prospect for biomedical field, especially for antibacteria, anticancer drug/gene delivery, cell imaging, biosensing, and so on [1].

Zinc oxide nanoparticles (ZnO NPs), as one of the most important metal oxide nanoparticles, are popularly employed in various fields due to their peculiar physical and chemical properties [2, 3]. ZnO NPs are firstly applied in the rubber industry as they can provide wearproof of the rubber composite, improve performance of high polymer in their toughness and intensity and antiaging, and other functions [4, 5]. Because of the strong UV absorption properties of ZnO, they are increasingly used in personal care products, such as cosmetics and sunscreen [6]. In addition, ZnO NPs have superior antibacterial, antimicrobial, and excellent UV-blocking properties. Therefore, in the textile industry, the finished fabrics by adding ZnO NPs exhibited the attractive functions of ultraviolet and visible light resistance, antibacteria, and deodorant [7]. Apart from the applications mentioned above, zinc oxide can also be used in other branches of industry, including concrete production, photocatalysis, electronics, electrotechnology industries, and so on [4, 8].

It is generally known that zinc as an essential trace element extensively exists in all body tissues, including the brain, muscle, bone, skin, and so on. As the main component of various enzyme systems, zinc takes part in body's metabolism and plays crucial roles in proteins and nucleic acid synthesis, hematopoiesis, and neurogenesis [2–5]. Nano-ZnO, with small particle size, makes zinc more easily to be absorbed by the body. Thus, nano-ZnO is commonly used as a food additive. Moreover, ZnO is graded as a “GRAS” (generally recognized as safe) substance by the US Food and Drug Administration (FDA) [9]. With these properties, ZnO NPs have received more attention in biomedical applications. Compared with other metal oxide NPs, ZnO NPs with the comparatively inexpensive and relatively less toxic property exhibit excellent biomedical applications, such as anticancer, drug delivery, antibacterial, and diabetes treatment; anti-inflammation; wound healing; and bioimaging [1, 10–12].

Herein, in this review, we will summarize the methods of synthesis and recent exciting progress on the use of ZnO NPs in the biomedical fields.

## 2. Synthesis of ZnO NPs

The biological activity of nanoparticles depends on factors including surface chemistry, size distribution, particle morphology, and particle reactivity in solution. Therefore, the development of nanoparticles with controlled structures that are uniform in size, morphology, and functionality is essential for various biomedical applications.

The ZnO NPs occurring in a very rich variety of size and shape will provide a wide range of properties. The methods for stable ZnO NPs preparation have been widely developed in recent years, which mainly include the chemical precipitation method, sol-gel method, solid-state pyrolytic method, solution-free mechanochemical method, and biosynthesis method.

### 2.1. Chemical Precipitation

The most popular method for ZnO NPs preparation is chemical precipitation, which usually involves two reaction reagents: a highly purified zinc forerunner such as zinc acetate (Zn(CH_3_COO)_2_·2H_2_O), zinc nitrate (Zn(NO_3_)_2_), or zinc sulfate (ZnSO_4_) and a solution of precipitator such as sodium hydroxide (NaOH) or ammonium hydroxide (NH_3_·H_2_O) [13]. Typically, the precipitator is added dropwise to the dissolved zinc precursor until the pH level reached about 10. Then, completely mix these solutions to get a white intermediate of zinc hydroxide. Ultimately, the sample of zinc hydroxide (Zn(OH)_2_) was converted to ZnO after sintering at high temperature.

Controlled parameters in this method mainly include the concentration of zinc forerunner and precipitator, the molar ratio of two reagents, and the reaction and calcination temperature.

Bisht et al. synthesized ZnO NPs using the chemical precipitation method using Zn(CH_3_COO)_2_·2H_2_O and NaOH in a molar ratio of 1 : 5. Intermediate products were calcined at 200°C for 2 h in a muffle furnace to obtain white fine powder ZnO with the size of 18.67 ± 2.2 nm [14].

Bettini et al. introduced a simplified precipitation approach in ZnO NPs using ZnSO_4_ and NaOH solution with a molar ratio of 1 : 2, which was carried out under vigorous stirring for 12 h at room temperature. The obtained white precipitate was washed several times and separated by centrifugation [15–17]. Finally, the precipitate (ZnO) was dried in an oven at 100°C for 6 h. The prepared ZnO NPs with a flake-like structure showed a size distribution about 100 nm.

The obtaining of ZnO NPs by the chemical precipitation method is not only simple and easily controlled but also easy industrialized. However, due to the surface effect of nanoparticles, the precursor of nanooxide prepared by the chemical precipitation method could form agglomerates easily.

### 2.2. Sol-Gel Method

A novel sol-gel synthesis of ZnO NPs was firstly presented by Spanhel and Anderson [18], which mainly involved three major steps:

1. Preparation of zinc precursor

A sample of Zn(CH_3_COO)_2_·2H_2_O was dissolved in ethanol, placed into a distillation apparatus, and then refluxed for few hours at atmospheric pressure. The solution was boiled nearly at 80°C and stirred to obtain the condensate and hygroscopic reaction mixture.

(2) Preparation of ZnO clusters

The hygroscopic mixture was diluted to ethanolic solution with the addition of LiOH·H_2_O powder. The suspension will become transparent with the help of an ultrasonic bath. This procedure could accelerate the release of OH ions, and the reaction at low temperature under air conditions could prevent rapid particle growth and get ZnO sols.

(3) Crystal growth

Crystal growth is a self-induced procedure occurring at room temperature. But the amount of LiOH which could strongly affect the crystals growth rate, shape, and size should be well controlled. LiOH-induced ZnO growth can be briefly summarized as follows:

(1) $$\begin{array}{c} \text{Zn}{\left({\text{CH}}_{3}\text{COO}\right)}_{2}\cdot 2{\text{H}}_{2}\text{O}+2\text{LiOH}⟶\text{Zn}{\left(\text{OH}\right)}_{2}+\;2{\text{CH}}_{3}\text{COOLi}+2{\text{H}}_{2}\text{O}\\ \text{Zn}{\left(\text{OH}\right)}_{2}+2{\text{H}}_{2}\text{O}⟶\text{Zn}{{\left(\text{OH}\right)}_{4}}^{2-}+2{\text{H}}^{+}\\ \text{Zn}{{\left(\text{OH}\right)}_{4}}^{2-}↔\text{ZnO}+{\text{H}}_{2}\text{O}+2{\text{OH}}^{-} \end{array}$$

Some other alkalis can also be used for ZnO growth; for instance, Rani et al. synthesized ZnO NPs using NaOH instead of LiOH and successfully obtained ZnO NPs with the largest crystallite size of 14 nm at 9 pH value [19].

The sol-gel method is the subject of much interest, in view of the simplicity, low cost, and relatively mild conditions of synthesis, which could offer a simple route to quantum size ZnO particles.

### 2.3. Solid-State Pyrolytic Method

The solid-state pyrolytic method was first developed by Wang et al. with advantages of low cost and easy operation for the growth of high-quality ZnO nanoparticles [20].

The typical synthesis procedure is as follows: Zn(CH_3_COO)_2_·2H_2_O and NaHCO_3_ are mixed at room temperature. The mixture is pyrolysed at the reaction temperature. Zn(CH_3_COO)_2_·2H_2_O is converted into ZnO, while NaHCO_3_ is transformed into CH_3_COONa and can be eventually cleaned out with deionized water. Subsequently, white ZnO NPs can be obtained via the thermal decomposition process. The particle sizes can be regulated by selecting different pyrolytic temperatures. Using this method, Wang et al. obtained ZnO NPs of different sizes in the range of 8–35 nm.

### 2.4. Solution-Free Mechanochemical Method

Solution-free mechanochemical preparation of ZnO NPs is a two-step synthesis method. The first step is to grind Zn(CH_3_COO)_2_ and H_2_C_2_O_4_·2H_2_O powder mixtures for a certain time to form ZnC_2_O_4_·2H_2_O nanoparticles [21].

Zn(CH_3_COO)_2_ (solid, large particles) + H_2_C_2_O_4_·2H_2_O(solid, large particles) = ZnC_2_O_4_·2H_2_O (solid particles) + 2CH_3_COOH (liquid and gas) + H_2_C_2_O_4_·2H_2_O (solid particles).

The second step is the thermal decomposition of ZnC_2_O_4_·2H_2_O nanoparticles at a very high temperature to get ZnO NPs:

(2) $$\begin{array}{c} {\text{ZnC}}_{2}{\text{O}}_{4}\cdot 2{\text{H}}_{2}\text{O}={\text{ZnC}}_{2}{\text{O}}_{4}+2{\text{H}}_{2}\text{O}↑\\ {\text{ZnC}}_{2}{\text{O}}_{4}\cdot 2{\text{H}}_{2}\text{O}=\text{ZnO}+{\text{CO}}_{2}↑+\text{CO}↑+2{\text{H}}_{2}\text{O}↑\\ \text{CO}+{\text{O}}_{2}={\text{CO}}_{2}↑\\ 2{\text{H}}_{2}{\text{C}}_{2}{\text{O}}_{4}\cdot 2{\text{H}}_{2}\text{O}+{\text{O}}_{2}=4{\text{CO}}_{2}↑+4{\text{H}}_{2}\text{O}↑\\ {\text{CH}}_{3}\text{COOH}\;\;\left(\text{liquid}\right)={\text{CH}}_{3}\text{COOH}\;\;\left(\text{gas}\right)↑ \end{array}$$

The advantages of this method are the low production costs and high homogeneity of the crystalline structure and morphology. But the morphology of the ZnO NPs strongly depends on the milling time of the reactant mixture, a longer time of milling led to a smaller particle size. The obtained ZnO NPs show an average size ranging from 24 to 40 nm.

Pardeshi and Patil synthesized ZnO NPs with different morphologies and crystallite sizes using this method by varying the calcination temperature from 400°C to 900°C. It was found that zinc oxide calcined from 400°C to 550°C exhibited the same crystallite growth rate (38–50 nm) [22].

### 2.5. Biological Methods

Physical and chemical methods for ZnO NPs preparations have widely developed. Nowadays, the development of green chemistry has attracted more and more attention because it is mostly environmentally friendly [23]. A broad variety of plant extract are used for the biosynthesis of ZnO NPs such as the leaf of *Azadirachta indica* (L.) [23], *Cochlospermum religiosum* (L.) [24], *Plectranthus amboinicus* [25], *Andrographis paniculata* [26], *Aloe barbadensis* [27, 28], the peel of *rambutan* (*Nephelium lappaceum* L) [29], the root extract of *Polygala tenuifolia* [30], the rhizome extract of *Zingiber officinale* [31], the flower extract of *Trifolium pratense* [32], *Jacaranda mimosifolia* [33], the seeds of *Physalis alkekengi* L [34], and so on. Biosynthetic and environment friendly technology for the synthesis of ZnO NPs are believed to be more ecofriendly, economical (low priced), nontoxic, and biocompatible than chemical and physical methods. ZnO NPs prepared by this method exhibited strong potential for biomedical applications such as its excellent anticancer and antibacterial activity.

## 3. Biomedical Applications of ZnO Nanoparticles

ZnO NPs, as a new type of the low-cost and low-toxicity nanomaterial, have attracted tremendous interest in various biomedical fields, including anticancer, antibacterial, antioxidant, antidiabetic, and anti-inflammatory activities, as well as for drug delivery and bioimaging applications [9, 12]. Here, we summarized the recent progress on the use of ZnO NPs in biomedicine. ZnO NPs less than 100 nm are considered to be relatively biocompatible, which support their biomedical applications and represent a powerful property in promoting the biomedical research.

### 3.1. Anticancer Activity

Cancer, a condition of uncontrolled malignant cell proliferation, is typically treated by chemotherapy, radiotherapy, and surgery in the past several decades. Although all these therapies seem to be very effective for killing cancer cells in theory, these nonselective therapy methods also introduce a lot of serious side effects [35]. Recently, nanomaterial-based nanomedicine, with high biocompatibility, easily surface functionalization, cancer targeting, and drug delivery capacity, has demonstrated the potential to overcome these side effects. Zn^2+^ is an essential nutrient for adults, and ZnO nanomaterials are considered to be safe in vivo. Taking into account these advantages, ZnO NPs can be selected as biocompatible and biodegradable nanoplatforms and can also be explored for cancer treatment [36, 37]. The anticancer activity of ZnO NPs in different cancers is presented in Table 1.

#### 3.1.1. Anticancer Activity by Inducing Cancer Cell Apoptosis

The mitochondrial electron transport chain is known to be associated with intracellular ROS generation, and anticancer agents entering into cancer cells could destroy the electron transport chain and release huge amounts of ROS [58, 59]. However, excessive ROS will lead to mitochondrial damage and result in the loss of protein activity balance that finally causes cell apoptosis [60]. ZnO NPs present certain cytotoxicity in cancer cells mainly by themselves based on a higher intracellular release of dissolved zinc ions, followed by increased ROS induction and induced cancer cell death via the apoptosis signaling pathway.

**Figure 1:**
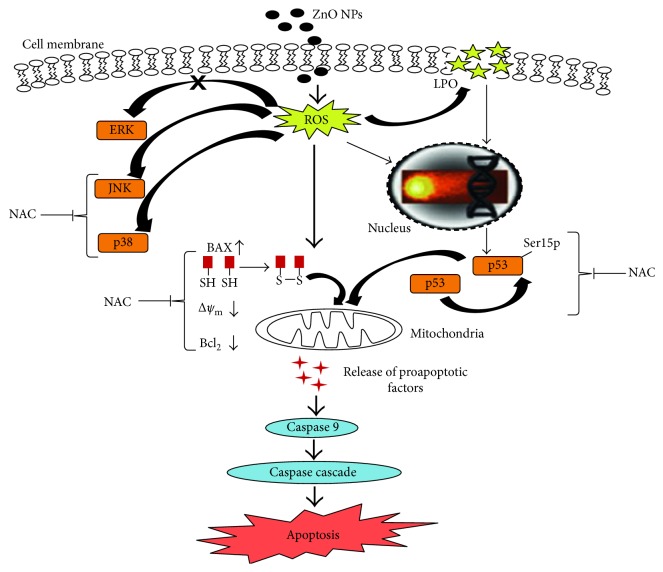
The mechanism of ZnO NPs-induced toxicity in human liver cells [42]. Copyright 2012 *Apoptosis*.

Sharma et al. explored the effects of ZnO NPs on human liver cancer HepG2 cells and its possible pharmacological mechanism [42]. ZnO NPs-exposed HepG2 cells presented higher cytotoxicity and genotoxicity, which were associated with cell apoptosis mediated by the ROS triggered mitochondrial pathway. The loss of the mitochondrial membrane potential could open outer membrane pores which would result in the release of some related apoptotic proteins including cytochrome c into the cytosol and activate the caspase. Mechanistic studies had proved that the loss of mitochondrial membrane potential-mediated HepG2 cell apoptosis was mainly due to the decrease in mitochondrial membrane potential and Bcl-2/Bax ratios as well as accompanying with the activation of caspase-9. Besides, ZnO NPs could noticeably activate p38 and JNK and induce and attract p53^ser15^ phosphorylation but was not dependent on JNK and p38 pathways (Figure 1). These results afforded valuable insights into the mechanism of ZnO NPs-induced apoptosis in human liver HepG2 cells.

Moghaddam et al. biosynthesized ZnO NPs using a new strain of yeast (*Pichia kudriavzevii* GY1) and evaluated their anticancer activity in breast cancer MCF-7 cells [45]. ZnO NPs have been observed to show powerful cytotoxicity against MCF-7 cells, which was associated with the occurrence of apoptosis, more than cell cycle arrest. The ZnO NPs-induced apoptosis was mainly through both extrinsic and intrinsic apoptotic pathways, and some antiapoptotic genes of Bcl-2, AKT1, and JERK/2 were downregulated, while proapoptotic genes of p21, p53, JNK, and Bax were upregulated.

ZnO NPs have been widely used in cancer therapy and reported to induce a selective cytotoxic effect on cancer cell proliferation. Chandrasekaran and Pandurangan investigated the cytotoxicity of ZnO nanoparticles against cocultured C2C12 myoblastoma cancer cells and 3T3-L1 adipocytes, which showed that ZnO NPs could be more cytotoxic to C2C12 myoblastoma cancer cells than 3T3-L1 cells. Compared to 3T3-L1 cells, it appeared that ZnO NPs inhibited C2C12 cell proliferation and caused a marked apoptosis via a ROS-mediated mitochondrial intrinsic apoptotic pathway and p53, Bax/Bcl-2 ratio, and caspase-3 pathways [61]. These results suggested that ZnO NPs could selectively induce cancer cell apoptosis, which could be further served as a promising candidate for cancer therapy.

#### 3.1.2. Anticancer by Autophagy

Autophagy is a highly regulated catabolic process that activated in response to different kinds of stresses like damaged organelles, ROS, anticancer agents, and protein aggregation. Excessive cellular damage may lead to cell death by the extension of autophagy and cellular self-consumption and result in cancer cell apoptosis [62, 63]. Hence, autophagy not only promotes cell survival but also activates lethal mechanisms in cancer cells, thus be considered as an important event in nanoparticle-induced cytotoxicity.

Bai et al. found that ZnO NPs with a crystal size of 20 nm resulted in a concentration-dependent loss of ovarian cancer SKOV3 cell viability [51]. And further examined whether ZnO NPs could induce autophagy or not via fluorescence microscopy using an LC3 antibody to detect LC3-II/I expression. Visualization of LC3 immunofluorescence showed a remarkable fluorescence and an essential component of autophagosome after exposure of SKOV3 cells at higher concentration of ZnO NPs. In addition, ZnO NPs-treated SKOV3 cells resulted in an upregulation of LC3-I/II and p53 expression, which further induced autophagic cell death.

Arakha et al. fabricated ZnO NPs using the chemical precipitation method and further evaluated their anticancer activity [64], which found that ZnO NPs with different sizes could obviously inhibit the proliferation of fibrosarcoma HT1080 cells. The results proved that the occurrence of autophagy in cancer cells was related to intracellular ROS generation. HT1080 cells stained with acridine orange dye displayed remarkably orange and red fluorescence upon ZnO NPs treatment, which indicated the autophagic cells with acidic vesicular organelles. Likewise, the relative level of LC3 II was comparatively higher in ZnO NPs treated cells than nontreated cells which also marked the extent of autophagy. Interaction ZnO NPs with HT1080 cell has relatively higher ROS generation. Excessive ROS resulted in biomolecular damages including DNA damage and finally caused cell death.

Previous studies have indicated that ROS and autophagy are involved in the cytotoxicity of ZnO NPs, but the regulatory mechanisms between autophagy and ROS remain to be elucidated. Zhang et al. investigated the regulatory mechanism of autophagy and the link between autophagy and ROS in ZnO NPs-treated lung epithelial cells [65]. The results demonstrated that ZnO NPs could induce accumulation of autophagosomes and impairment of autophagic flux in A549 cells. This autophagy induction was positively correlated with the dissolution of ZnO NPs in lysosomes to release zinc ions, and zinc ions released from ZnO NPs were able to damage lysosomes, leading to impaired autophagic flux and mitochondria. Impaired autophagic flux resulted in the accumulation of damaged mitochondria, which could generate excessive ROS to cause cell death. This research provided a novel insight into the regulation mechanisms of autophagy-lysosomes-mitochondria-ROS axis, which would contribute to a better understanding of the toxicity of nanomaterials.

#### 3.1.3. Anticancer Drug Delivery

Using nanoparticles in targeted drug delivery provides exciting opportunities for much more safety and effective cancer treatment. By targeting the specific sites of cancer cells, nanoparticle-based drug delivery could reduce the overall amount of drugs used and thus minimize undesirable side effects [9, 66]. Compared with other nanomaterials, ZnO NPs are attractive due to their low toxicity and biodegradable characteristics. ZnO NPs have acquired tremendous interest in cancer drug delivery. Different types of drugs such as doxorubicin, paclitaxel, curcumin, and baicalin or DNA fragments could be loaded onto the ZnO NPs to show better solubility, higher toxicity compared with individual agents, and effective delivery into cancer cells [48, 67–69].

Hariharan et al. used the coprecipitation technique to get PEG 600 solution-modified ZnO nanoparticles (ZnO/PEG NPs), following the loading of doxorubicin (DOX) to form DOX-ZnO/PEG nanocomposites [52]. DOX-ZnO/PEG nanocomposites not only enhanced the intracellular accumulation of DOX but also presented a concentration-dependent inhibition on cervical cancer HeLa cell proliferation. Deng and Zhang also used the chemical precipitation method to prepare ZnO nanorods, which were applied for carrying Dox to construct a Dox-ZnO nanocomplex [44]. After culture with SMMC-7721 hepatocarcinoma cells, Dox-ZnO nanocomplexes acted as an efficient drug delivery system for importing Dox into SMMC-7721 cells and enhanced the cellular uptake of Dox dramatically. Furthermore, coupled with ultraviolet (UV) illumination, Dox-ZnO nanocomplexes caused more cell death through photocatalytic properties and synergistically triggered caspase-dependent apoptosis.

Puvvada et al. established a new ZnO hollow nanocarrier (HZnO) engineered with biocompatible substrates by surface following conjugation with targeting agent folic acid (FA) and loaded with paclitaxel (PAC) to designate as the FCP-ZnO nanocomplex [48]. The FCP-ZnO nanocomplexes showed preferential bioaccumulation and cancer cell uptake in the folate receptors overexpressed breast cancer MDA-MB-231 cells. Because of FA-mediated endocytosis and intracellular release within the acidic endolysosome, the FCP-ZnO nanocomplexes not only exhibited significantly higher cytotoxicity in vitro MDA-MB-231 cells but also reduced MDA-MB-231 xenograft tumours in nude mice.

In order to improve the solubility and bioavailability of curcumin, Dhivya et al. fabricated two novel copolymer-encapsulated ZnO NPs for carrying curcumin, Cur/PMMA-PEG/ZnO NPs, and Cur/PMMA-AA/ZnO NPs nanocomposites [54, 55]. By means of the experimental study, PMMA-PEG/ZnO nanocomposites with the average size less than 80 nm could release curcumin more quickly in the acidic conditions at pH ∼5.8. Compared to constituent nanomaterials (nanocurcumin, PMMA-PEG, ZnO NPs, and PMMA-PEG/ZnO), the Cur/PMMA-PEG/ZnO nanocomposite performed largest observable inhibition on human gastric cancer AGS cell viability (IC_50_ ∼0.01 *μ*g/mL^−1^) and induced cell cycle arrest at the S phase. For another nanocomposite, PMMA-AA/ZnO NPs with a size of 42 ± 5 nm could carry a large amount of curcumin and also had obvious antiproliferation on AGS cancer cells.

#### 3.1.4. Targeting Functionalization

Targeted nanoparticles (NPs) also provide more therapeutic benefits besides specificity and specific localization like high payload, multidrug conjugation, easy tuning of release kinetics, selective localization, and bypass of multidrug resistance mechanism [70]. In order to increase the targeting effects and selectivity against cancer cells, plenty of functionalization techniques have been reported for nanoparticle modification. Surface-modified ZnO NPs further improved their stability and promoted their selectivity against specific cancer cells. The central attention is on the functionalization of the ZnO NPs surface with different kinds of biological molecules comprising different types of proteins, peptides, nucleic acids, folic acid, hyaluronan, and so on [47, 57, 71–73]. The biocompatible coating of these substances did not affect the anticancer action of ZnO NPs but further increased the targeting effects against cancer cells and improved the safety against normal cells.

For example, Chakraborti et al. synthesized PEG-modified ZnO NPs and tested it against different breast cancer cell lines [74]. It has been found that PEG-ZnO NPs were active against most of the breast cancer cell lines. The main mechanism by which PEG-ZnO kills a cancer cell is by generating ROS and triggering p53-dependent apoptosis leading to cell death.

Namvar et al. produced hyaluronan/ZnO nanocomposite (HA/ZnO) through green synthesis for the first time for cancer treatment [57]. The HA/ZnO nanocomposites caused morphological changes and inhibited proliferation of cancer cells (pancreatic adenocarcinoma PANC-1 cell, ovarian adenocarcinoma CaOV-3 cell, colonic adenocarcinoma COLO205 cell, and acute promyelocytic leukemia HL-60 cell) in dose- and time-dependent manner. Encouraging, HA/ZnO nanocomposite treatment for 72 hours did not cause toxicity to the normal human lung fibroblast (MRC-5) cell line. Compared to bare ZnO NPs, RGD peptide modification also increased the targeting effects of ZnO NPs on integrin *α*v*β*3 receptors overexpressed MDA-MB-231 cells [47]. It appeared to increase the toxicity of the ZnO NPs to breast cancer MCF-7 and MDA-MB-231 cells at lower doses.

In general, the anticancer activity of nanoscaled ZnO materials with prominent functionality may provide a new opportunity for exploiting ZnO NPs in treating cancer diseases. The theory analysis and experimental research proved that ZnO NPs with less side effect present greater selectivity among normal and cancerous cells. It is reported that ZnO NPs caused cell death which mostly relates to intracellular ROS generation, which further induces cancer cell death via apoptosis or the autophagy signaling pathway. But up to now, the advanced anticancer mechanism study of ZnO NPs is still lacked of, especially in cellular and molecular mechanism strengthening. Therefore, in subsequent research work, we should attach more importance to their molecular mechanism in vitro and vivo and overcome its limitations in cancer therapy.

### 3.2. Antibacterial Activity

**Figure 2:**
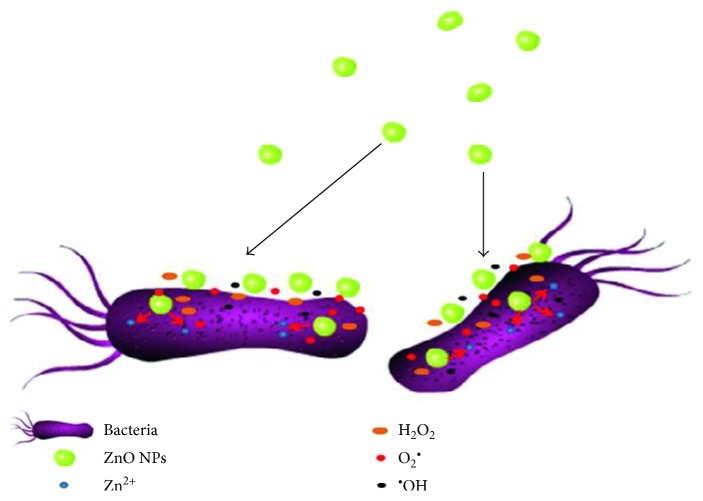
Schematic illustration of antibacterial activity of ZnO NPs.

ZnO NPs can be selected as an antibacterial material because of its superior properties, such as high specific surface area and high activity to block a wide scope of pathogenic agents. But recently, the antibacterial activity of ZnO NPs is still scarcely known. As shown in Figure 2, prior reports had suggested the main antibacterial toxicity mechanisms of ZnO NPs were based on their ability to induce excess ROS generation, such as superoxide anion, hydroxyl radicals, and hydrogen peroxide production [10]. The antibacterial activity may involve the accumulation of ZnO NPs in the outer membrane or cytoplasm of bacterial cells and trigger Zn^2+^ release, which would cause bacterial cell membrane disintegration, membrane protein damage, and genomic instability, resulting in the death of bacterial cells [75–77].

Presently, Gram-negative *Escherichia coli* (*E. coli*) and Gram-positive *Staphylococcus aureus* (*S. aureus*) are mainly chosen as model bacteria to evaluate the antibacterial activity of ZnO NPs [77, 78]. Some other Gram-negative bacteria such as *Pseudomonas aeruginosa* (*P. aeruginosa*) [24, 79], *Proteus vulgaris* (*P. vulgaris*) [80], *Vibrio cholerae* (*V. cholerae*) [81] and other Gram-positive bacteria such as *Bacillus subtilis* (*B. subtilis*) [82] and *Enterococcus faecalis* (*E. faecalis*) [83] are also investigated. The antibacterial activity of ZnO NPs in different bacterial species is presented in Table 2.

Jiang et al. reported the potential antibacterial mechanisms of ZnO NPs against *E. coli* [76]. It showed that ZnO NPs with an average size about 30 nm caused cell death by directly contacting with the phospholipid bilayer of the membrane, destroying the membrane integrity. The addition of radical scavengers such as mannitol, vitamin E, and glutathione could block the bactericidal action of ZnO NPs, potentially revealing that ROS production played a necessary function in the antibacterial properties of ZnO NPs. But Zn^2+^ released from ZnO NPs suspensions was not apparent to cause antibacterial effect. Reddy prepared ZnO NPs with sizes of ∼13 nm and examined their antibacterial (*E. coli* and *S. aureus*) activities [78]. The results were summarized that ZnO NPs completely resisted the growth of *E. coli* at concentrations of about 3.4 mM but inhibited growth of *S. aureus* at much lower concentrations (≥1 mM). Moreover, Ohira and Yamamoto also found the antibacterial (*E. coli* and *S. aureus*) activity of ZnO NPs with small crystallite sizes was stronger than those with large crystallite sizes [97]. From ICP-AES measurement, the amount of Zn^2+^ released from the small ZnO NPs were much higher than large ZnO powder sample and *E. coli* was more sensitive to Zn^2+^ than *S. aureus*. So we can believe that eluted Zn^2+^ from ZnO NPs also take a key role in antibacterial action.

Iswarya et al. extracted crustacean immune molecule *β*-1,3-glucan binding protein (Ph*β*-GBP) from the heamolymph of *Paratelphusa hydrodromus* and then successfully fabricated the Ph*β*-GBP-coated ZnO NPs. The Ph*β*-GBP-ZnO NPs were spherical in shape with a particle size of 20–50 nm and restrained the growth of *S. aureus* and *P. vulgaris*. It should be noted that *S. aureus* was more susceptible to Ph*β*-GBP-ZnO NPs than *P. vulgaris*. In addition, Ph*β*-GBP-ZnO NPs could alter cell membrane permeability and trigger high level of ROS formation both in *S. aureus* and *P. vulgaris* [87]. Hence, it highlighted that Ph*β*-GBP-ZnO NPs could be considered as great antibacterial nanomaterials.

Epidemic disease *cholera*, a serious diarrheal disease caused by the intestinal infection of Gram-negative bacterium *V. cholera*, mainly affects populations in the developing countries [81, 94]. Aiming at the development of nanomedicine against *cholera*, Sarwar et al. carried out a detailed study about ZnO NPs against *Vibrio cholerae* (two biotypes of cholera bacteria (classical and El Tor)). ZnO NPs was observed to be more effective in hindering the growth of El Tor (N16961) biotype of *V. cholera*, which was closely associated with ROS production. These results would damage bacterial membrane, increase permeabilization, and substantially modify their morphology [85]. They also detected the antibacterial activity of the ZnO NPs in cholera toxin (CT) mouse models. It was found that ZnO NPs could induce the CT secondary structure collapsed gradually and interact with CT by interrupting CT binding with the GM1 anglioside receptor [98].

Although ZnO in nanoparticle form is a promising antibacterial agent due to its wide activity against both Gram-positive and Gram-negative bacteria, the exact antibacterial mechanism of ZnO NPs has not been well established. Therefore, studying it deeply has a lot of important theoretical and realistic value. In the future, we believe ZnO NPs can be explored as antibacterial agents, such as ointments, lotions, and mouthwashes. In addition, it can be coated on various substrates to prevent bacteria from adhering, spreading, and breeding in medical devices.

### 3.3. ZnO NPs for Diabetes Treatment

Diabetes mellitus is a serious public health problem, and the WHO has estimated that, in 2014, there were more than 400 million adults with diabetes all over the world [99]. Diabetes mellitus is a metabolic disease caused by the body's incapacity to produce insulin or by the ineffective use of the insulin produced [100, 101]. Zinc is a trace element and abundantly found mineral in all human tissues and tissue fluids. Zinc is well known to keep the structural integrity of insulin and has an active role in the secretion of insulin from pancreatic cells. It also participates in insulin synthesis, storage, and secretion [102]. Therefore, ZnO NPs as a novel agent in order for zinc delivery have been developed and evaluated for their antidiabetic potential.

Kitture et al. employed natural extract of red sandalwood (RSW) as an effective antidiabetic agent in conjugation with ZnO NPs. The antidiabetic activity was assessed with the help of *α*-amylase and *α*-glucosidase inhibition assay with murine pancreatic and small intestinal extracts [103]. Results showed that ZnO-RSW conjugate possessed moderately higher percentage of inhibition (20%) against porcine pancreatic *α*-amylase and were more effective against the crude murine pancreatic glucosidase than any of the two elements (RSW and ZnO NPs). The conjugated ZnO-RSW displayed 61.93% of inhibition in glucosidase while the bare ZnO NPs and RSW showed 21.48% and 5.90%, respectively.

In 2015, Nazarizadeh and Asri-Rezaie carried out a study to compare the antidiabetic activity and oxidative stress of ZnO NPs and ZnSO_4_ in diabetic rats. It found that ZnO NPs with small dimensions at higher doses (3 and 10 mg/kg) had a much greater antidiabetic effect compared to ZnSO_4_ (30 mg/kg). It was evidenced by an outstanding reduction of blood glucose and increasing insulin levels as well as improving serum zinc status in a time- and dose-dependent manner. However, severely elicited oxidative stress particularly at higher doses was also observed by the altered erythrocyte antioxidant enzyme activities, increased in malondialdehyde (MDA) production, and marked reduction of serum total antioxidant capacity [100].

The hyperglycemia can directly enhance an inflammatory state by regulating C-reactive protein (CRP) and cytokines, such as interleukins, which is involved in the development of cardiovascular diseases. Hussein et al. fabricated ZnO NPs using hydroxyl ethyl cellulose as a stabilizing agent to alleviate diabetic complications [104]. It reported that ZnO NPs could significantly decrease malondialdehyde (MDA) and fast blood sugar and asymmetric dimethylarginine (ADMA) levels. The inflammatory markers, interleukin-1 (IL-1*α*) and CRP, were also notably decreased after ZnO NPs treatment, concomitant with an increase in nitric oxide (NO) and serum antioxidant enzyme (PON-1) levels in diabetic rats.

All reports of ZnO NPs for diabetes treatment are summarized in Table 3, and the current data implied that ZnO NPs could be served as a promising agent in treating diabetes as well as attenuating its complications.

### 3.4. Anti-Inflammatory Activity

Inflammation is part of the complex biological response of body tissues to harmful stimuli, such as pathogens, damaged cells, or irritants [111]. Since the advent of nanoparticles and considering these biological activities of zinc ions, the anti-inflammatory effects of ZnO NPs have also attracted much attention.

Atopic dermatitis (AD) is a chronic inflammatory skin disease characterized by the impairment of the skin-barrier functions, which was involved with complex interaction between genetic and environmental factors [112, 113]. Textiles have the longest and most intense contact with the human skin. Wiegand explored the role of ZnO-functionalized textile fibers in the control of oxidative stress in AD in vitro and in vivo [114]. The study found that it is an obvious improvement of AD pruritus and subjective sleep quality when AD patients wore the ZnO textiles overnight on 3 consecutive days. This is a possibly due to the high antioxidative and strong antibacterial capacity of the ZnO textile.

Ilves et al. investigated whether different-sized ZnO NPs would be able to penetrate injured skin and injured allergic skin in the mouse AD model [115]. Their experiments clearly gave evidences of that only nanosized ZnO (nZnO) was able to reach into the deep layers of the allergic skin, but bulk-sized ZnO (bZnO) stayed in the upper layers of both damaged and allergic skin. Compared with bZnO, nZnO exerted higher anti-inflammatory properties by decreasing drastically on proinflammatory cytokines (IL-10, IL-13, IFN-*γ*, and Th2 cytokines) in the mouse model of AD. These results demonstrated that ZnO NPs with a small size had great effects on reducing skin inflammation in AD models.

The anti-inflammatory activity of ZnO NPs is not confined to atopic dermatitis treatment but has also shown to be very effective for other inflammatory diseases. Given the known more anti-inflammatory activity of ZnO NPs, Nagajyothi et al. described a straightforward, inexpensive, and ecofriendly ZnO NPs using the root extract of *P. tenuifolia* and the anti-inflammatory activities were investigated in LPS-stimulated RAW 264.7 macrophages [30]. ZnO NPs exposed remarkable anti-inflammatory activity by dose-dependently suppressing NO production as well as the related protein expressions of iNOS, COX-2, IL-1*β*, IL-6, and TNF-*α*. Thatoi et al. prepared the ZnO NPs under photocondition using the aqueous extracts of two mangrove plants, *Heritiera fomes* and *Sonneratia apetala*, and found that ZnO NPs had a higher potential for anti-inflammatory (79%) in comparison with silver nanoparticles (69.1%) [116].

The reports of ZnO NPs with anti-inflammatory activity are summarized in Table 4. Hence, ZnO NPs also have the potential to be utilized for anti-inflammatory treatment.

### 3.5. ZnO NPs for Bioimaging

ZnO NPs exhibit efficient blue emissions and near-UV emissions, which have green or yellow luminescence related to oxygen vacancies, therefore further extending its application into bioimaging field [12, 36, 120].

**Figure 3:**
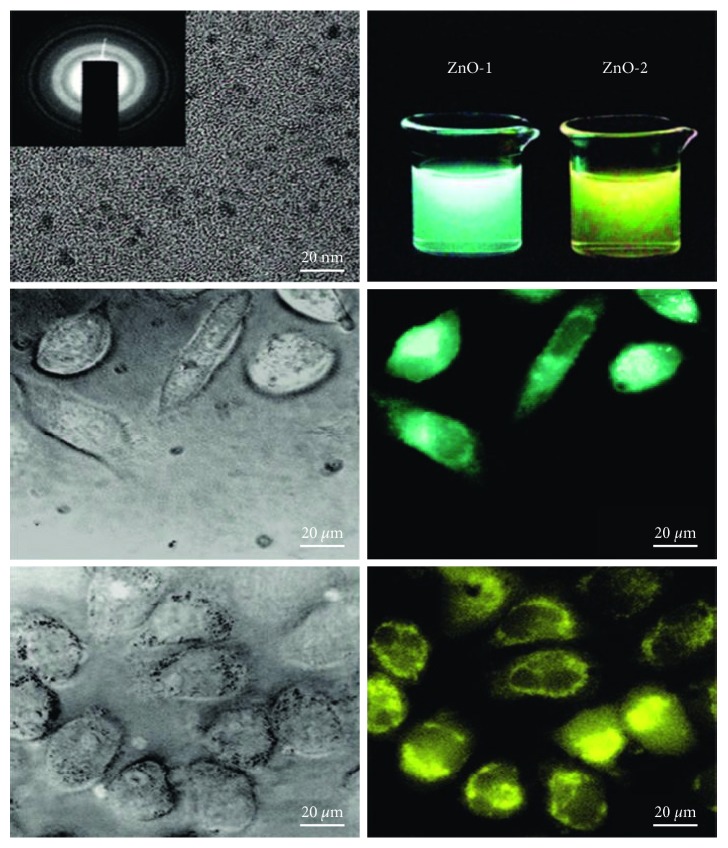
The upper part is the high-resolution transmission electron microscopy (HRTEM) image of the ZnO@polymer core-shell nanoparticles and the aqueous solutions of ZnO-1 and ZnO-2 under a UV light; the middle part is the DIC picture and the fluorescent image of the human hepatoma cells labeled by ZnO-1; and the lower part is the DIC picture and the fluorescent image of the hepatoma cells labeled by ZnO-2 [121]. Copyright 2008 American Chemical Society.

Using a simple sol-gel method, Xiong et al. prepared stable aqueous ZnO@polymer core-shell nanoparticles (ZnO@poly(MAA-co-PEGMEMA))for the first time. The ZnO@polymer core-shell nanoparticles exhibited high quantum yield and very stable broad photoluminescence in aqueous solutions. As shown in Figure 3, within human hepatoma cells, ZnO-1 (derived from LiOH) with an average size of 3 nm showed green fluorescence, while ZnO-2 (derived from NaOH) with an average size of 4 nm appeared yellow. It was worth to note that these nanoparticles did not show any remarkable toxicity for human hepatoma cells when their concentrations were less than 0.2 mg/mL. Furthermore, the luminescence was very stable during cell culturing and the cells were alive at 45 min of exposure. So, as a type of safe and cheap luminescent labels, the ZnO@polymer core-shell nanoparticles can be used as fluorescent probes for cell imaging in vitro [121].

Jiang et al. constructed ZnO nanosheets for the imaging of cultured cells. They treated drug sensitive leukemia line K562 cells with ZnO nanosheets, and the yellow-orange light emission was clearly observed around or inside the cells under UV irradiation (365 nm) at room temperature [122]. ZnO nanostructures were successfully attached onto or penetrated into the cells, which suggested that ZnO nanosheets with visible yellow-orange emission could act as a feasible label for the bioimaging.

Tang et al. prepared ZnO NPs by using the chemical precipitation method. It exhibited emission colors of blue, green, yellow, and orange [123]. The emission color can be changed via adjusting the pH of the precipitation solutions. To stabilize ZnO NPs in water, they encapsulated the ZnO NPs with silica to form ZnO@silica core-shell nanostructures. The obtained ZnO@silica core-shell nanoparticles exhibited excellent water stability, and the visible emissions of ZnO were retained. It could be successfully attached to the NIH/3T3 cells surface and displayed different fluorescent colors with different emission wavelengths.

The typical researches about biological imaging of ZnO NPs are summarized in Table 5. Based on its advanced intrinsic fluorescence, ZnO nanomaterial can also be used as a promising candidate for cell imaging and pathological studies.

## 4. Conclusions and Future Perspectives

ZnO NPs have exhibited promising biomedical applications based on its anticancer, antibacterial, antidiabetic, anti-inflammatory, drug delivery, as well as bioimaging activity. Due to inherent toxicity of ZnO NPs, they possess strong inhibition effects against cancerous cell and bacteria, by inducing intracellular ROS generation and activating apoptotic signaling pathway, which makes ZnO NPs a potential candidate as anticancer and antibacterial agents. In addition, ZnO NPs have also been well known to promote the bioavailability of therapeutic drugs or biomolecules when functioning as drug carriers to achieve enhanced therapy efficiency. Moreover, with the ability to decrease blood glucose and increase in insulin levels, ZnO NPs have shown the promising potential in treating diabetes and attenuating its complications, which can be further evaluated.

ZnO NPs are listed as a kind of safe substance by the FDA. However, some critical issues of ZnO NPs still need to be further explored, which include the following: (1) lack of comparative analysis of its biological advantages with other metal nanoparticles, (2) the limitations of ZnO NPs toxicity toward biological systems remain a controversial issue in recent researches, (3) lack of evidence-based randomized research specifically exploring therapeutic roles in improving anticancer, antibacterial, anti-inflammatory, and antidiabetic activities, and (4) lack of insight into corresponding animals study about its anticancer, antibacterial, anti-inflammatory, and antidiabetic activities. Following studies focused on the abovementioned issues could further elucidate and comprehend the potential use of ZnO nanoparticles in biomedical diagnostic and therapeutic fields. We believe that nanomaterials would dramatically promote the development of medicine, and ZnO nanoparticles are expected to make more exciting contributions in these fields.

## Figures and Tables

**Table 1:** The anticancer effects of ZnO NPs in different human cancer cell lines.

Cancer type	Effect and mechanism
Colon cancer	ZnO NPs suppressed cell viability in Caco-2 cell line via increased ROS and induced IL-8 release [38]
	ZnO NPs and fatty acids could induce lysosomal destabilization in Caco-2 cells [39]
	ZnO NPs induced Caco-2 cells cytotoxicity associated with increased intracellular Zn ions [40]
	ZnO NPs conjugated with peptides had a higher antiproliferation in HT-29 colon cancer cells than other Au NPs and Fe_3_O_4_ NPs [41]

Hepatocarcinoma	ZnO NPs caused ROS generation and oxidative DNA damage and lead to mitochondrial-mediated apoptosis in HepG2 cells [42]
	ZnO NPs selectively induce apoptosis in HepG2 cells, which was also mediated by ROS via the p53 pathway [43]
	Dox-ZnO nanocomplex can act as a drug delivery system to increase the internalization of the anticancer drug Dox in SMMC-7721 cells [44]

Breast cancer	Ecofriendly formulated ZnO NPs arrest the cell cycle in the G2/M phase and upregulated proapoptotic genes p53, p21, Bax, and JNK and downregulated antiapoptotic genes Bcl-2, AKT1, and ERK1/2 in a dose-dependent manner in MCF-7 cells [45]
	A doxorubicin delivery system based on zinc oxide nanomaterials can bypass the P-gp increase in the drug accumulation in resistant MCF-7R and MCF-7S cells [46]
	RGD (Arg-Gly-Asp)-targeted ZnO NPs can target integrin *α*v*β*3 receptors to increase the toxicity of the ZnO NPs to MDA-MB-231 cells at lower doses [47]
	ZnO-Fe_3_O_4_ magnetic composite nanoparticles site-specific have no significant toxicity towards noncancerous NIH 3T3 cells but show obvious toxicity at similar concentration to MDA-MB-231 cells [14]
	FA-functionalized PTX-ZnO NPs released ∼75% of the paclitaxel payload within six hours in acidic pH, improved chemotherapy tolerance, and increased antitumor efficacy [48]

Lung cancer	ZnO NPs incorporated in liposomes not only rendered pH responsivity to the delivery carrier but also exhibited synergetic chemo-photodynamic anticancer action [49]
	Human lung adenocarcinoma cells with an EGFR mutation are sensitive to ZnO NP20 and Al-ZnO NP20, which resulted in nonautophagic cell death [50]

Ovarian cancer	ZnO NPs are able to induce significant cytotoxicity, apoptosis, and autophagy in SKOV3 cells through reactive oxygen species generation and oxidative stress [51]

Cervical cancer	DOX-ZnO/PEG nanocomposites exhibited better dose-dependent toxicity towards HeLa cell lines [52]
	ZnO nanoparticles showed a dynamic cytotoxic effect in cervical carcinoma cells which induced the apoptosis through the increased intracellular ROS level and upregulated apoptotic gene p53 and caspase-3 expression [53]
Gastric cancer	PMMA-AA/ZnO NPs and PMMA-PEG/ZnO were able to carry a large amount of the hydrophobic drug (curcumin) showing highly anti-gastric cancer activity [54, 55]

Human epidermal cancer	ZnO NPs induce cell death at high concentrations, and at lower concentrations, they induce cell cycle arrest in the S and G 2/M phase by intracellular ROS generation in A431 cells [56]

Acute promyelocytic leukemia	HA/ZnO nanocomposite caused G2/M cell cycle arrest and stimulated apoptosis-related increase in caspase-3 and −7 activities of the HL-60 cells [57]

**Table 2:** The antibacterial effects of ZnO NPs in different bacterial species.

Material	Size (nm)	Bacterial species	Antibacterial mechanism
ZnO NPs	30	*E. coli*	Destroy the membrane integrity and ROS production [76]
Ag-ZnO composite	64	*S. aureus* and GFP *E. coli*	ROS and the release of Ag^+^ and Zn^2+^ [84]
ZnO NPs	∼80	*V. cholera*	Depolarization of the membrane structure, increased permeabilization and damage of DNA, and generation of ROS [85]
ZnO NPs	∼20	*E. coli* 11634	Hydrogen peroxide (H_2_O_2_) [86]
ZnO NPs		*E. coli* and *S. aureus*	Release of Zn^2+^ [78]
Ph*β*-GBP-coated ZnO NPs (Ph*β*-GBP-ZnO NPs)	20∼50	*S. aureus* and *P. vulgaris*	Alter the bacterial cell membrane permeability and high level of ROS [87]
ZnO nanocatalyst	∼18	*B. subtilis*, *E. coli, K. pneumonia*, and *S. typhimurium*	H_2_O_2_, OH^−^, and other ROS [88]
CdO-ZnO nanocomposite	27	*E. coli*, *P. aeruginosa*, *Klebsiella pneumonia*, *S. aureus*, *P. vulgaris*, and *Bacillus* spp	ROS (OH^−^, H_2_O_2_, and O_2_^2−^) and the release of Zn^2+^ and Cd^2+^ [89]
ZnO quantum dots	4	*E. coli* MG1655, *Cupriavidus metallidurans* CH34	The toxicity is mainly from Zn^2+^ [90]
ZnO/kaoline nanocomposites		*S. aureus*, *E. coli*, *E. faecalis*, and *P. aeruginosa*	Zn^2+^ and consequent diffusion of these ions into the cytoplasm [91]
ZnO nanostructures (ZnO-NSs)	70∼80	*S. aureus*, *S. typhimurium*, *P. vulgaris*, and *K. pneumoniae*	ROS damage to cell membranes [92]
ZnO NPs	40	*Streptococcus mutans* (MTCC497), *S. pyogenes* (MTCC1926), *Vibrio cholerae* (MTCC3906), *Shigella flexneri* (MTCC1457), and *Salmonella typhi* (MTCC1252)	ROS and the release of Zn^2+^ [93]
ZnO NPs	90∼100	*V. cholera* and enterotoxic *E. coli* (ETEC)	Inhibit adenylyl cyclase activity, and cAMP levels are decreased [94]
Ge-ZnO NPs	20	*P. aeruginosa* and *E. faecalis*	Penetrated the cell and caused bacterial cell death [83]
SA/ZnO composites		*E. coli* and *S. aureus*	ROS production [95]
ZnO@GA NPs	11.5 ± 4.4	*E. coli* and *S. aureus*	Attributed to the high affinity of GA for the bacterial cell membrane and the increased lipophilicity upon the addition of GA [96]

**Table 3:** ZnO NPs for diabetes treatment.

Type of NPs	Size	Drug loaded/synergy	Effects
ZnO NPs	60–95 nm spherical	—	Mitigated the diabetic complications [104]
ZnO NPs	∼20 nm spherical	—	A significant decrease in fasting blood glucose and increase in high-density lipoprotein levels [105]
ZnO NPs	10–30 nm	Thiamine	ZnO NPs in combination with thiamine-improved diabetes therapy [106]
ZnO NPs	—	—	ZnO NPs effectively reversed diabetes-induced pancreatic injury [107]
ZnO-RSW NPs	∼20 nm	Conjugated red sandalwood (RSW)	ZnO-RSW NPs showed excellent activity against the crude murine pancreatic glucosidase as compared to the individual ZnO NPs and the RSW extract [103]
ZnO NPs	—	—	ZnO NPs acted as a potent antidiabetic agent evidenced by improved glucose disposal, insulin levels, and zinc status in diabetic rats [100]
ZnO NPs	∼10 nm	—	ZnO NPs presented pleiotropic antidiabetic effects via improved insulin signaling, enhanced glucose uptake, decreased hepatic glucose output, decreased lipolysis, and enhanced pancreatic beta cell mass [108]
ZnO NPs, CeO_2_ NPs, Ag NPs	ZnO NPs: 55 nm, CeO_2_ NPs: 54 nm, Ag NPs: 22.5 nm	—	ZnO NPs and Ag NPs had more potent antihyperglycemic activity than CeO_2_ NPs [109]
ZnO NPs	Spherical: 96–115 nm; hexagonal: 57 ± 0.3 nm	—	ZnO NPs displayed better antidiabetic potential (IC_50_: 66.78 *μ*g/mL) than ZnNO_3_ (IC_50_: 91.33 *μ*g/mL) in terms of the α-amylase inhibition activity [26]
ZnO NPs	—	Vildagliptin	ZnO NPs and vildagliptin have synergistic effects on the therapy of type-2 diabetes [110]
ZnO NPs	10–15 nm spherical	—	ZnO NPs could improve glucose tolerance and higher serum insulin and reduce blood glucose, nonesterified fatty acids, and triglycerides [101]

**Table 4:** ZnO NPs with anti-inflammatory activity.

Type of NPs	Size	Effects
ZnO-functionalized textile fibers	—	A rapid improvement of AD severity, pruritus, and subjective sleep quality when AD patients wore the ZnO textiles overnight on 3 consecutive days [114]
ZnO NPs	—	ZnO NPs exerted higher anti-inflammatory properties by decreasing drastically on proinflammatory cytokines in the mouse model of AD [115]
ZnO NPs	Spherical: 33.03–73.48 nm	ZnO NPs relieved inflammation and displayed a dose-dependent effect in the suppression of related protein expressions [30]
ZnO NPs	Spherical: 96–115 nm; hexagonal: 57 ± 0.3 nm	ZnO NPs exhibited excellent anti-inflammatory activity with 66.78 *μ*g/mL IC_50_ value [26]
ZnO incorporated into TiO_2_ nanotubes (TNTs/ZnO)	—	TNTs/ZnO had a significant inhibitory effect on the proliferation and adhesion of macrophages [117]
ZnO NPs and Ag NPs	—	ZnO NPs had a higher potential for anti-inflammatory (79%) in comparison with Ag NPs (69.1%) [116]
ZnO NPs	69.4 ± 13.0 nm	The anti-inflammatory abilities of ZnO NPs to suppress proinflammatory cytokines IL-1*β* and TNF-*α* and myeloperoxidase (MPO) in the colitic mice and activate Nrf2 signaling [118]
ZnO NPs	—	Dietary supplementation with ZnO NPs was effective in inhibiting mRNA expression of inflammatory cytokines (IFN-*γ*, IL-1*β*, TNF-*α*, and NF-*κ*B) in the ileum in weaned piglets [119]

**Table 5:** Typical researches about biological imaging of ZnO NPs.

Materials	Size and models	Biological imaging
ZnO@MAA-co-PEGMEMA	Human hepatoma cells	With tunable photoluminescence emission and high quantum yield, under UV light, ZnO-1 showed green fluorescence, while ZnO-2 appeared yellow [121]
ZnO@PMAA-co-PDMAEMA	Spherical: 4 nm/COS-7 cells	The PDMAEMA-modified ZnO QDs emitted strong yellow luminescence under UV light [124]
ZnO-Au@PEG NPs	<100 nm/B16F10 cells	ZnO-Au@PEG NPs can penetrate into the living cells and exhibit bright yellow fluorescence [125]
ZnO nanosheets	46.6 ± 8.5 nm/K562 cells	Yellow-orange light emission was observed around or inside the cells under UV light [122]
ZnO@silica NPs	2.7–4.4 nm/HeLa cells	Monodispersed ZnO@silica NPs with blue, green, and yellow emission through using VTES, TEOS, and APS as modification materials [126]
Fe_3_O_4_-ZnO NPs	15.7 nm/dendritic cells	Fe_3_O_4_-ZnO NPs emit green fluorescence under UV light irradiation [127].
ZnO@silica NPs	NIH/3T3 cells	ZnO@silica NPs exhibited emission colors of blue, green, yellow, and orange under 365 nm excitation via the adjustment of the pH of the precipitation solutions [123]
ZnO@silica NPs	55 nm/nerve cells, Caco-2 cells	ZnO@silica QD colloidal solution exhibited a significant blue emission [128]
ZnO nanowires	20–50 nm/U87MG cells, MCF-7 cells	ZnO nanowires exhibited green fluorescent. RGD peptide-conjugated green fluorescent ZnO NWs can be specifically targeted to cell surface receptors in vitro [129]
